# Long-term functional and structural outcomes in X-linked retinoschisis: implications for clinical trials

**DOI:** 10.3389/fmed.2023.1204095

**Published:** 2023-06-15

**Authors:** Beau J. Fenner, Jonathan F. Russell, Arlene V. Drack, Alina V. Dumitrescu, Elliott H. Sohn, Stephen R. Russell, H. Culver Boldt, Louisa M. Affatigato, Jeremy M. Hoffmann, Jeaneen L. Andorf, Edwin M. Stone, Ian C. Han

**Affiliations:** ^1^Institute for Vision Research, University of Iowa, Iowa City, IA, United States; ^2^Department of Ophthalmology and Visual Sciences, Carver College of Medicine, University of Iowa, Iowa City, IA, United States; ^3^Department of Medical Retina, Singapore National Eye Centre, Singapore, Singapore; ^4^Singapore Eye Research Institute, Singapore, Singapore; ^5^Ophthalmology and Visual Sciences Academic Clinical Program, Duke-NUS Graduate Medical School, Singapore, Singapore

**Keywords:** retina, genetics, inherited eye disease, retinoschisis, retinal detachment, gene therapy

## Abstract

**Introduction:**

X-linked retinoschisis (XLRS) is an inherited retinal disease (IRD) caused by pathogenic mutations in the retinoschisin gene, *RS1*. Affected individuals develop retinal layer separation, leading to loss of visual acuity (VA). Several XLRS gene therapy trials have been attempted but none have met their primary endpoints. An improved understanding of XLRS natural history and clinical outcomes may better inform future trials. Here, we report the long-term functional and structural outcomes of XLRS and the relevance of *RS1* genotypes to the visual prognosis of affected individuals.

**Methods:**

A retrospective chart review of patients with molecularly confirmed X-linked retinoschisis was performed. Functional and structural outcomes, and RS1 genotype data, were included for analysis.

**Results:**

Fifty-two patients with XLRS from 33 families were included in the study. Median age at symptom onset was 5 years (range 0–49) and median follow-up was 5.7 years (range 0.1–56.8). Macular retinoschisis occurred in 103 of 104 eyes (99.0%), while peripheral retinoschisis occurred in 48 of 104 eyes (46.2%), most often in the inferotemporal quadrant (40.4%). Initial and final VA were similar (logMAR 0.498 vs. 0.521; *p* = 0.203). Fifty of 54 eyes (92.6%) developed detectable outer retinal loss by age 20, and 29 of 66 eyes (43.9%) had focal or diffuse outer retinal atrophy (ORA) by age 40. ORA but not central subfield thickness (CST) was associated with reduced VA. Inter-eye correlation was modest for VA (*r*-squared = 0.03; *p* = 0.08) and CST (*r*-squared = 0.15; *p* = 0.001). Carbonic anhydrase inhibitors (CAIs) improved CST (*p* = 0.026), but not VA (*p* = 0.380). Eight of 104 eyes (7.7%) had XLRS-related retinal detachment (RD), which was associated with poorer outcomes compared to eyes without RD (median final VA 0.875 vs. 0.487; *p* <0.0001). *RS1* null genotypes had greater odds of at least moderate visual impairment at final follow-up (OR 7.81; 95% CI 2.17, 28.10; *p* = 0.002) which was independent of age at onset, initial CST, initial ORA, or previous RD.

**Discussion:**

Overall, long-term follow-up of XLRS patients demonstrated relatively stable VA, with presenting CST, development of ORA, and null *RS1* mutations associated with poorer long-term visual outcomes, indicating a clinically relevant genotype-phenotype correlation in XLRS.

## 1. Introduction

In the past decade, inherited retinal diseases (IRDs) have become the most common cause of legal blindness among working age adults in several developed nations (1, 2), having overtaken diabetic retinopathy with the advent of anti-vascular endothelial growth factor (VEGF) agents and improved community screening. X-linked retinoschisis (XLRS) is a relatively uncommon IRD, with a population prevalence estimated between 1 to 200 cases per 100,000 (3–5). XLRS is characterized by separation of the neurosensory retinal layers, often during infancy or childhood (“juvenile” XLRS), resulting in schisis cavities that cause reduced central vision and relative scotomata (6, 7). Reduced structural integrity of the retina may also lead to vitreous hemorrhage, full-thickness retinal breaks, and/or retinal detachment (RD) (8). Strabismus has also been observed in children with deprivation amblyopia secondary to macular schisis (9, 10) or axial hyperopia (11).

The tendency of XLRS to affect the pediatric population and poor visual outcomes due to schisis-related RD has led to considerable interest in development of gene therapies (12–17). XLRS is caused by pathologic mutations in the *RS1* gene, which encodes retinoschisin, a 224 amino acid extracellular lectin that binds retinal cell plasma membranes and enables cell-to-cell adhesion (18–21). With a coding sequence of only 675 nucleotides (22), full-length *RS1* is easily packaged into conventional adeno-associated viral (AAV) vectors for gene augmentation. However, *RS1* gene therapy clinical trials have thus far failed to meet their primary endpoints (14, 23), perhaps due to short time points (e.g., 9 months) of study as well as clinically significant intraocular inflammation that contributed to study discontinuation. As such, long-term functional and structural data to aid in clinical trial design as well as inform patient selection for future studies will increase the likelihood of success of any emerging gene replacement strategies.

Despite the availability of data from several large XLRS clinical cohort studies (24–30), the optimal therapeutic trial design for this disease remains unclear. More specifically, changes in visual acuity over the natural history of disease, relationships between macular schisis severity and visual function, and the relevance of *RS1* genotypes to visual prognosis must be clearly understood if demonstrable efficacy of a XLRS gene- or cell-based therapy is sought. In the current work, we performed a retrospective study of the functional, structural, and genetic outcomes of a large cohort of molecularly confirmed XLRS patients from the United States, with the primary aim to demonstrate long-term structural and functional outcomes to better inform current therapeutic approaches, especially clinical trial design.

## 2. Materials and methods

### 2.1. Study design and ethics statement

We retrospectively analyzed clinical records of patients with molecularly confirmed XLRS. All patients provided written consent to participate in the study, which was approved by the institutional review board of the University of Iowa and was conducted in accordance with the tenets of the Declaration of Helsinki on Biomedical Research Involving Human Subjects.

### 2.2. Patients

**Figure 1:**
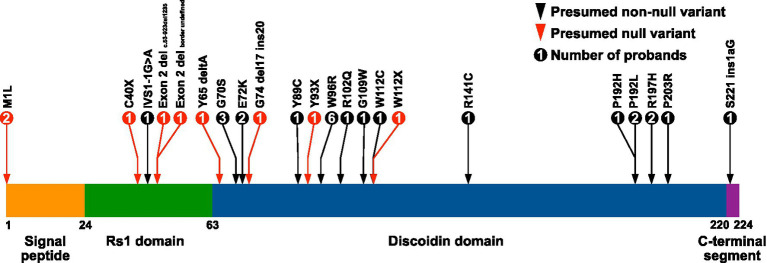
Locations of *RS1* variants in the XLRS study cohort. The amino acid positions of the retinoschisin domain junctions are indicated below the diagram. Retinoschisin domain structure adapted from Bush et al. (32). Exon 2 deletions were c.53–923del1235 and a second with an undefined border.

Patients included in the study were examined in the Department of Ophthalmology at the University of Iowa from 1964 to 2022. The clinical diagnosis of XLRS was made by an IRD specialist based on clinical history, examination by slit lamp and indirect biomicroscopy, and available ancillary testing, which variably included multimodal imaging and electroretinography. Each patient included in the study had molecular confirmation of pathologic variants in *RS1* through the John and Marcia Carver Nonprofit Genetic Testing Laboratory (Institute for Vision Research, University of Iowa, Iowa City, IA). *RS1* variants treated as null for the purposes of statistical analyses were based on previous work characterizing the functional domains and *in vitro* behavior of retinoschisin mutants (20, 31). The 3′ *RS1* variant S221ins1aG was located beyond the C-terminal end of the central discoidin domain of retinoschisin and was not considered null for the purposes of this study (Figure 1).

### 2.3. Visual acuity

Best corrected visual acuity was determined using subjective refraction with Snellen or appropriate equivalent pediatric acuity charts. Acuities were converted to logMAR decimal values for the purposes of statistical analysis using previously described methods (33). Clinical records were reviewed to identify the age when the patient stated their visual symptoms began (i.e., became symptomatic), prior to which the patient was considered presymptomatic.

### 2.4. Optical coherence tomography analysis

Macular optical coherence tomography (OCT) data were acquired using the Spectralis HRA + OCT instrument (Heidelberg Engineering Inc., Franklin, Massachusetts) with volume scan protocols incorporating either 19- or 61-line scans per eye. Several patients were initially imaged using a time domain Stratus OCT instrument (Carl-Zeiss Meditec, Dublin, CA), so these data were only used for gross feature examination and not for quantitative analyses. Centration of the ETDRS grid overlay and retinal layer segmentation were corrected manually when required using the Heidelberg Eye Explorer software version 1.9. Central subfield thickness (CST; central 1 mm zone of ETDRS grid overlay) data were collected for each patient at baseline and at their final clinical visit. Patients presenting prior to the availability of Spectralis HRA + OCT were not included in the OCT analysis component of the study.

The extent of macular retinoschisis was scored by noting the presence of retinoschisis (or the sequela of outer retinal atrophy) in the central 1 mm, 3 mm, or 6 mm zones of the ETDRS grid overlay, paralleling macular zones of XLRS involvement as previously described (34). Foveal outer retinal atrophy (ORA) was scored using a four-step scale based on similar prior analysis (30), with grade 0 representing intact ellipsoid zone (EZ) and RPE, grade 1 being visibly increased lucency of the EZ with or without increased lucency of the RPE within the central 1 mm zone. For grade 1, well-demarcated hypotransmission artefacts due to overlying schisis cavities were not considered to represent ORA. Grade 2 was defined as localized or focal areas of EZ disruption with or without adjacent RPE disruption in the central 1 mm zone, and grade 3 being diffuse EZ disruption with or without diffuse RPE disruption in the central 1 mm zone. These grades corresponded to the spectrum of structural changes observed in the XLRS cohort and were considered of sufficient range and specificity to represent the progression of disease.

### 2.5. Longitudinal data and treatment

Visual acuity and OCT data as above were recorded at baseline and at the last available follow up for each patient. Commencement and cessation dates for CAI use were recorded. In all cases, the CAI used was brinzolamide 1% or dorzolamide 2%, given to the treated eye two or three times daily. Only cases where patient compliance during the treatment period was documented were used for analysis. The presence of peripheral schisis was recorded for each patient and visit, and the anatomic location was noted (superotemporal, superonasal, inferonasal, and inferotemporal quadrants) based on clinical notes including fundus drawings as well as available fundus photography. Patients who developed retinal detachment (RD) were scored, including dates and types of treatment (e.g., repair with pars plana vitrectomy) and number of surgeries.

### 2.6. Statistical analyses

Trendlines for visual acuity and CST data were estimated using third order polynomial regression. Differences in visual acuity between initial and final visits, rates of CST change, and responses to topical CAI treatment were compared using Wilcoxon matched pairs signed rank testing. For CAI treatment, CST at the commencement of treatment was recorded for the treated eye, and the CST at between 3 to 6 months (the earliest date was taken) after commencement. Differences in visual acuity outcomes and outer retinal atrophy grades were compared using Kruskal–Wallis testing. Inter-eye correlation for visual acuity and CST were determined using linear regression. Comparisons of visual acuity outcomes between RD and no RD patients were made using Mann Whitney testing. For all statistical testing, a *p*-value of less than 0.05 was considered statistically significant. Prism version 9 (GraphPad Software, Boston, Massachusetts) was used to perform all statistical analyses.

Multivariable regression was performed using categorical derivatives of clinical and genetic outcome data. Specifically, age at symptom onset was classified as <5 or ≥5 years, CST as <450 μm or ≥450 μm, ORA grade as <2 or ≥2, presence of *RS1* null variants, and VA at final clinic visit of <0.5 or ≥0.5 logMAR units. Category cutoffs were derived from median values for the relevant variable (age of symptom onset, CST, VA at final visit, ORA grade), or from the presence of *RS1* null variants. Odds ratios were adjusted for age at final clinic visit in all cases, and were additionally adjusted for the remaining categorical variables, with or without adjustment for presence of previous retinal detachment. Multivariable regression analyses were performed using Wizard version 2.0 (Evan Miller Software).

## 3. Results

### 3.1. Presentation of disease and functional outcomes

**Figure 2:**
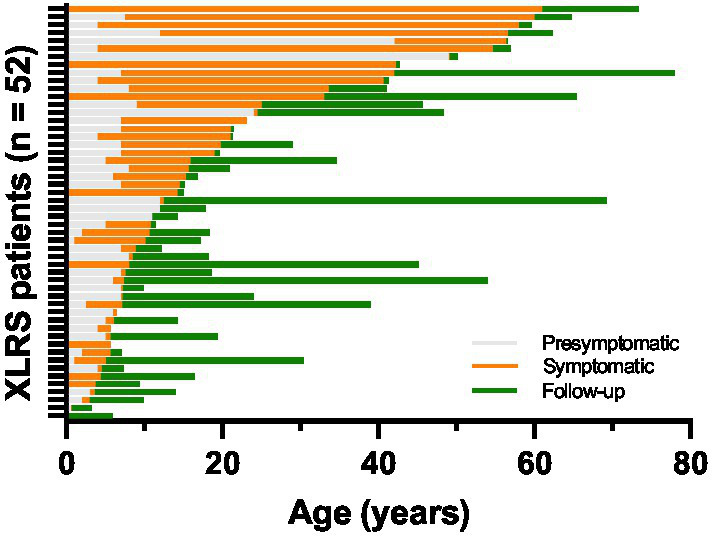
Duration of symptoms and clinical follow-up for the XLRS cohort (*n* = 52). Individual horizontal bars represent time periods for individual patients. The presymptomatic period (gray bars) represents the time prior to a patient’s stated onset of symptoms, while the symptomatic period (orange bars) represents time after onset of symptoms but prior to presentation for clinical evaluation. The period during which clinical data was available for analysis is represented by green bars.

**Figure 3:**
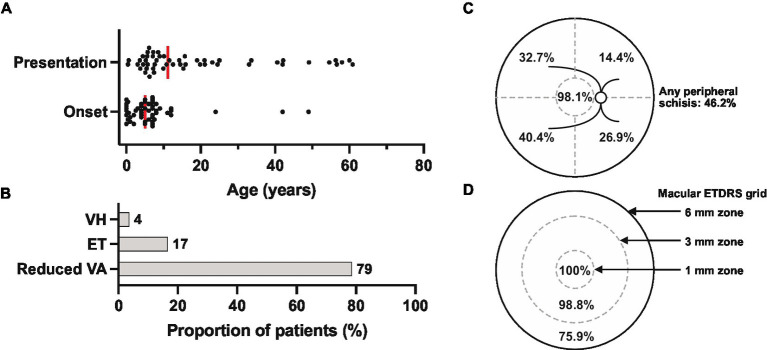
Presenting features of the XLRS cohort (*n* = 52 patients). **(A)** Reported age of symptom onset (median = 5) and patient age at presentation (median = 11). Each dot represents one patient, and medians are represented by the vertical red bars. **(B)** Presenting symptoms. VH, vitreous hemorrhage; ET, esotropia; VA, visual acuity. **(C)** Summary of the locations of retinoschisis (macula or superotemporal, superonasal, inferonasal, and inferotemporal quadrants) identified by clinical biomicroscopy at presentation (*n* = 104 eyes). **(D)** Extent of macular involvement by optical coherence tomography (*n* = 83 eyes), with the central circle representing the central 1 mm zone centered on the fovea. ETRDS, early treatment of diabetic retinopathy study standard grid.

Clinical records were reviewed for a total of 52 patients from 33 families evaluated at the University of Iowa between 1964 and 2022. The age and duration of symptom onset and clinical follow-up for each patient is displayed in Figure 2. Median age at symptom onset was 5 and median age at presentation was 12 (Figure 3A), with the most common presenting symptom being reduced central vision (41/52; Figure 3B), followed by esotropia (9/52), and floaters from vitreous hemorrhage (2/52). Patients were followed for a median duration of 5.8 years (range 0–56.8). On clinical examination, all but two of 104 eyes (98.1%) were noted to have macular involvement (Figures 3C,D), with peripheral involvement in 46.2% of eyes which was localized to the following quadrants: inferotemporal, 40.4%; superotemporal, 32.7%; inferonasal, 26.9%; and superonasal, 14.4%.

**Figure 4:**
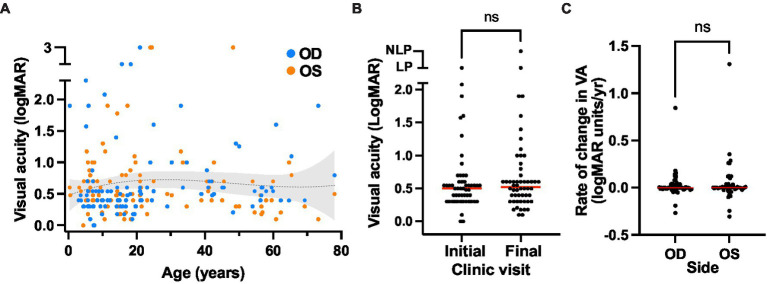
Visual acuity outcomes of the XLRS cohort. **(A)** Visual acuity over time. The presenting and final visual acuities is plotted over age, with a trendline (third order polynomial, *r*-squared = 0.01) with 95% confidence interval overlay. **(B)** Comparison of visual acuity at the initial (median VA OD = 0.498 logMAR units, or 20/63 Snellen) and final visit (median VA 0.521 logMAR units, or 20/66 Snellen). No significant difference was observed using a Wilcoxon matched pairs signed rank test (*p* = 0.203). **(C)** Rate of change in visual acuity over time in the XLRS cohort. Red bars indicate the median rates of visual acuity change for the right and left eyes. No significant difference was observed between eyes using a Wilcoxon matched pairs signed rank test (*p* = 0.567).

Visual acuity in most patients remained relatively stable even over prolonged clinical follow-up (Figure 4A). The median age at first recorded VA was 10.6 (range 0.4–60.9) and at final recorded VA was 21.0 (range 3.2–78.0), and no significant difference was found between the initial and final VA values for the cohort (Figure 4B). Over a median follow-up duration of 7 years, the median annual change in logMAR visual acuity for all cases with follow-up data available (*n* = 49) was 0 with a range of −0.31 to 1.31 (mean 0.03 ± 0.17; mean letter loss of 1.57 ± 8.59/year; Figure 4C).

### 3.2. Structural outcomes of the XLRS cohort

**Figure 5:**
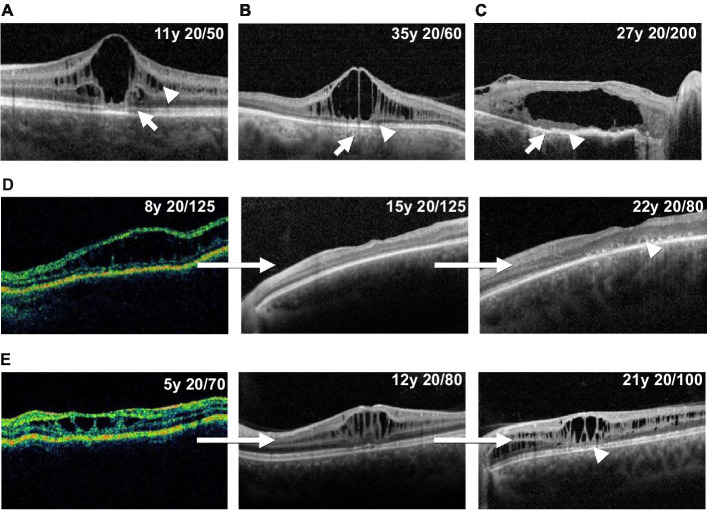
Illustrative optical coherence tomography findings in XLRS patients. Panels A, B and C show example outer retinal atrophy grades. **(A)** Increased ellipsoid lucency (grade 1, arrow) and inner nuclear layer schisis (arrowhead). **(B)** Hypertransmission defects (arrow) and associated focal ellipsoid disruption (grade 2, arrowhead). **(C)** Hypertransmission defects (arrow) and extensive loss of the ellipsoid band (grade 3, arrowhead). Panels **(D)** and **(E)** show example longitudinal cases. **(D)** Progression of macular schisis, with collapse of initial schitic spaces by age 15, and progression to outer retinal atrophy (arrowhead) by age 22. **(E)** Slow decline in visual acuity, relatively stable schisis, and eventual development of foveal outer retinal atrophy (arrowhead) over a 16 years observation period.

Macular OCT data were available for 83 of 104 XLRS eyes. Typical schisis of the inner nuclear layer was observed in most cases, with variable involvement of the outer plexiform layer (Figures 5A–E). Retinoschisis or resultant outer retinal atrophy (ORA) was present within the central 6 mm zone in 63/83 (75.9%) of eyes, the central 3 mm zone in 19/83 (22.9%) and was confined to only the central 1 mm zone in 1/83 (1.2%) of eyes. The extent of macular involvement remained stable over the follow-up duration for all but one eye, where there was spontaneous regression of schisis from the 6 mm zone to the 3 mm zone.

**Figure 6:**
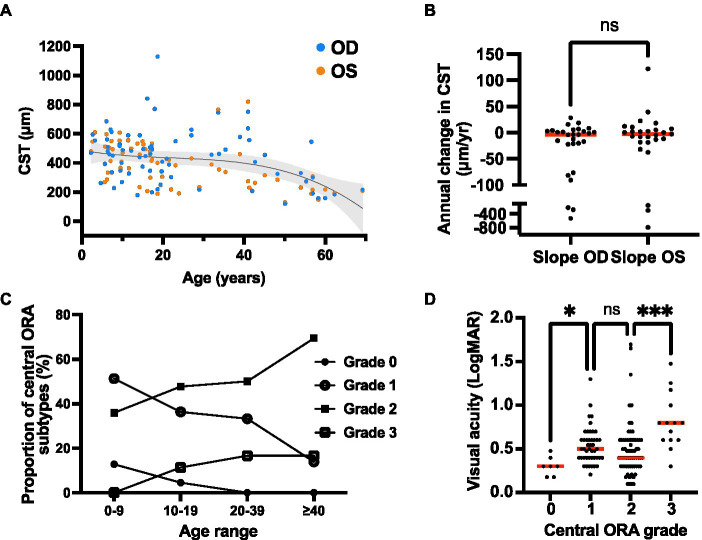
Quantitative optical coherence tomography (OCT) outcomes of the XRLS cohort. **(A)** Change in CST over time. The CST values are shown for each eye (*n* = 88 available) at the initial and final available scan, with a third-order polynomial regression line (*r*-squared = 0.181) and 95% confidence interval overlay plotted for both eyes. **(B)** Rate of change in CST over time for right and left eyes (*n* = 29 pairs). Red bars show median rates of change. Ns, no significant difference (Wilcoxon matched pairs signed rank test; *p* = 0.726). **(C)** Proportion of eyes with each grade of ORA within three different age categories. **(D)** Relationship between visual acuity and ORA grade in XLRS patients. Red bars show median visual acuity. ^*^Significant (Kruskal–Wallis test; *p* = 0.028); ns, not significant (*p* = 0.579); ^***^significant (*p* = 0.0004).

**Figure 7:**
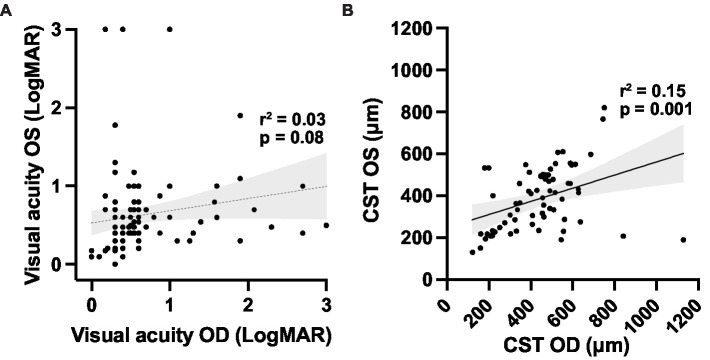
Correlations between **(A)** right and left eye visual acuity; and **(B)** macular optical coherence tomography outcomes for the XLRS cohort. Linear regression trendlines with 95% confidence interval bands are shown for each variable. CST, central subfield thickness.

The central subfield thickness (CST) remained relatively stable over the first four decades of life, followed by an apparent decline after age 40, consistent with interval atrophy (Figure 6A). Additionally, the CST showed relative intra- and inter-eye stability over time (Figure 6B). We also observed the appearance and progression of macular ORA in all cases with sufficient data by age 40 (Figure 6C), while 50% and 16.7% of cases had localized or near complete macular ORA, respectively. The extent of ORA was significantly associated with decreasing visual acuity (*p* = 0.028 for grade 0 vs. grade 1 ORA; *p* = 0.0004 for grade 2 vs. grade 3 ORA; Figure 6D), but no significant association was observed between visual acuity and CST (Supplementary Figure S1). We additionally examined the relationship between CST and ORA (Supplementary Figure S2) and observed that there was no significant difference in CST between eyes without ORA and ORA grades 1, 2, or 3. A significant difference was observed only between ORA grades 1 and 3, with grade 1 having greater CST than grade 3 (479 μm vs. 199 μm; *p* = 0.0036). Inter-eye visual acuity and CST measures were poorly correlated (Figure 7).

### 3.3. Treatment outcomes

**Figure 8:**
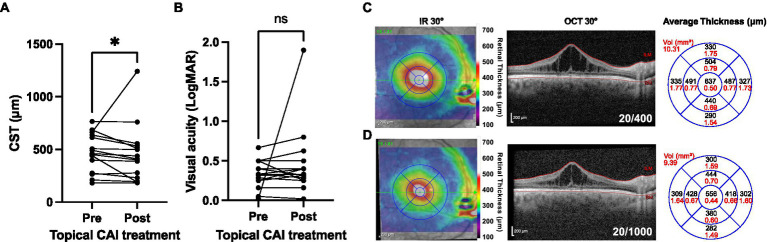
Structural and functional outcomes of topical carbonic anhydrase inhibitor (CAI) use in XRLS patients. **(A)** Macular central subfield thickness (CST) values at the time of CAI commencement and between 3–6 months after drug commencement (*n* = 15). **(B)** ^*^Significant (post < pre; Wilcoxon matched pairs signed rank test; *p* = 0.026). **(B)** Visual acuity outcomes for the eyes shown in panel **(A)**. Ns, not significant (Wilcoxon matched pairs signed rank test; *p* = 0.380). **(C,D)** Illustrative case showing the right eye macular OCT of a 41 years-old male with XLRS (genotype *RS1* G70S) at baseline **(C)** and after 3 months **(D)** of treatment with three times daily topical dorzolamide. Reduction of CST is observed but with decline in visual acuity and interval progression of underlying foveal ORA. Infra-red (IR) 30° images with retinal thickness heat map overlays are shown to the left, the 30° spectral domain OCT (segmented at the internal limiting membrane, ILM, and bruch membrane, BM) in the center, and the average retinal thickness and volume within ETDRS grid sectors to the right.

Topical CAIs were used for management of macular schisis at some point in 38 of 52 patients (73.1%) of patients, while an oral CAI (acetazolamide 250 or 500 mg daily) was used in only two (3.8%) patients, both of whom reported variable compliance. One of these patients stopped using the medication after 2 months due to systemic side effects (fatigue and neuropathy). Among patients managed with topical CAI, 17 of 38 (44.7%) were continued on medication for at least 1 year and were on medication at the time of their last clinic visit, while 11 of 38 (28.9%) stopped medication due to ocular side effects or unspecified noncompliance. The remaining 10 of 38 (26.3%) cases were commenced on topical CAIs at the time of their final clinic visit. Subsequent analysis was confined to patients using topical CAIs. Data were available for the immediate pretreatment period and the 3 to 6 months posttreatment period for 15 eyes (Figures 8A,B). A very small but significant reduction in CST occurred following CAI initiation [mean CST of 470 μm (range 182–766 μm) pretreatment vs. 454 μm (range 183–1,242 μm) posttreatment; *p* = 0.023], while no significant improvement in visual acuity was detected (mean VA 0.33 pretreatment vs. 0.45 posttreatment; *p* = 0.380; Figures 8C,D). The limited improvement in CST was due to a single outlier that worsened from 663 to 1,242 μm following treatment. Exclusion of this individual resulted in a mean improvement of 58.1 μm post-treatment (*p* = 0.001), but again no significant change in visual acuity was observed. Separation of the treated patients into pediatric (≤18 years; *n* = 9 eyes) and adult (>18 years; *n* = 6 eyes) subgroups showed that the CST reduction was significant in pediatric eyes (mean CST change of −82.2 ± 90.4 μm; *p* = 0.037) but not adult eyes (mean CST change −26.0 ± 31.3 μm; *p* = 0.098). No significant change in visual acuity was observed in the same age-based subgroup analysis, with logMAR VA changes of 0.02 ± 0.07 (*p* = 0.469) in the pediatric subgroup and 0.0005 ± 0.14 (*p* = 0.993) in the adult subgroup. At the individual eye level, the CST of 3/15 eyes worsened, 7/15 eyes improved by 1–50 μm, 3/15 eyes improved by 51–100 μm, and 2 eyes improved by >100 μm.

**Figure 9:**
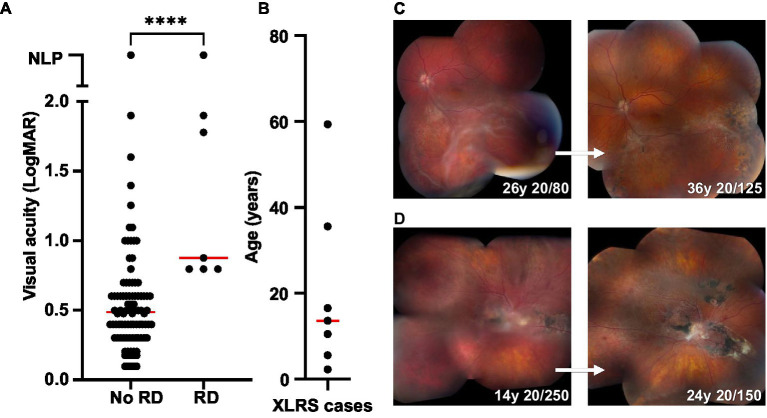
Retinal detachment (RD) outcomes in XLRS patients. **(A)** Comparison of final visual acuities for eyes with and without RD. ^****^Significant (median VA 0.487, *n* = 90 for no RD group; median VA 0.875, *n* = 7 for RD group; Mann Whitney test, *p* < 0.0001). **(B)** Age at time of RD surgery for XLRS patients (*n* = 7; median 13.6). **(C,D)** Montage color fundus photos showing the 10 years outcomes of two individuals with XLRS-related RD. **(C)** Left eye macula-splitting inferotemporal RD managed with scleral buckle, vitrectomy, endolaser barricade, and silicone oil, with subsequent oil removal and cataract extraction with intraocular lens placement. **(D)** Right eye macula-off RD managed with vitrectomy, membrane peeling, temporal retinectomy, endolaser, and silicone oil, with subsequent oil removal and cataract extraction with intraocular lens placement.

Among the 104 XLRS eyes in the study cohort, eight eyes (7.7%) from seven patients developed schisis-related RD (Figure 9). Peripheral schisis was present in all eyes that developed RD, compared to 42.1% of eyes without RD (chi-squared 9.84; *p* = 0.002). Five of eight cases had been on routine clinical follow-up prior to development of RD (range 1.4–27.5 years of follow-up) and returned with new symptoms, while three cases had undergone surgery prior to evaluation in our clinic. Of the three cases previously undergoing follow-up in our clinic, one retained the same vision pre- and postoperatively (20/125), one had mild worsening of vision (from 20/80 to 20/125) and the last worsened (20/300 to 20/1250). All cases were managed surgically with pars plana vitrectomy, with a median age at surgery of 12 (range 1.8 to 59.4 years). Additional surgery was performed for silicone oil removal (2/7) and for lensectomy and intraocular lens implantation (7/7). Final visual acuity (≥1 year post-operative) in eyes with RD was poorer than eyes without RD (median VA 0.875 vs. 0.487; *p* < 0.0001). Despite this, operated eyes generally retained stable anatomy with prolonged follow-up (Figures 9C,D), except for a single eye that developed intractable proliferative vitreoretinopathy and eventual phthisis.

### 3.4. Phenotype and genotype correlates in the XLRS cohort

Finally, we assessed the impact of phenotypic and genotypic findings on visual outcomes in XLRS patients. The visual outcome measure used was a VA of 0.5 logMAR units (Snellen 20/63) or worse, corresponding to the WHO visual impairment category of moderate (35). After adjustment for age as a possible confounder, increased odds of moderate visual impairment or worse occurred in eyes with presenting CST of ≥450 μm (OR 4.65; 95% CI 1.26, 17.09; *p* = 0.021) and with a *RS1* null genotype (OR 7.18; 95% CI 2.09, 24.64; *p* = 0.002). Results remained similar even after adjustment for previous retinal detachment (Table 1), indicating that these two variables are clinically useful prognostic indicators for visual outcomes in XLRS. Presence of ORA of at least grade 2 did not increase odds of moderate visual impairment in this analysis.

**Table 1:** Visual outcomes are robustly associated with genotype and presenting macular thickness in XLRS patients. Multivariable regression was performed with the outcome variable of moderate visual impairment (35) or worse (logMAR visual acuity 0.5 or Snellen 20/63; *n* = 47/104) at final follow-up.

Explanatory variable	*n*	Model 1		Model 2		Model 3	
		Odds ratio (95% CI)	Significance	Odds ratio (95% CI)	Significance	Odds ratio (95% CI)	Significance
Symptom onset before age 5	44/104	3.15 (1.36, 7.28)	0.007^a^	2.68 (0.88, 8.15)	0.081	2.57 (0.79, 8.63)	0.116
Initial CST ≥450 μm	39/81	3.96 (1.21, 12.88)	0.022^a^	4.65 (1.26, 17.09)	0.021^a^	4.51 (1.17, 17.38)	0.029^a^
Initial ORA grade of >1	44/83	0.90 (0.35, 2.31)	0.835	0.64 (0.20, 1.97)	0.438	0.59 (0.18, 1.93)	0.388
*RS1* null genotype^b^	26/104	3.65 (1.39, 9.55)	0.008^a^	7.18 (2.09, 24.64)	0.002^a^	7.81 (2.17, 28.10)	0.002^a^

Multivariable regression was performed with the outcome variable of moderate visual impairment (35) or worse (logMAR visual acuity 0.5 or Snellen 20/63; *n* = 47/104) at final follow-up.

a Statistical significance with *p* < 0.05. Model 1 adjusted for age at final clinic visit (continuous variable), model 2 adjusted for age at final clinic visit and remaining categorical variables, and model 3 adjusted for age at final clinic visit, remaining categorical variables, and previous retinal detachment.

b Presumed *RS1* null genotypes are indicated in Figure 1.

Repeat analysis of the cohort after exclusion of eyes with RD yielded the same association between visual outcome and a null *RS1* genotype (OR 7.81; 95% CI 2.17, 28.10; *p* = 0.002). Development of RD was more common among eyes with null *RS1* genotypes, but this did not reach statistical significance (37.5% vs. 23.9%; chi-squared 0.715; *p* = 0.397). Severity of ORA at final clinic visit was also assessed as an outcome measure: a significantly higher proportion of eyes with *RS1* null genotypes had extensive ORA at the final clinic follow-up compared to eyes with non-null genotypes (36% vs. 3%; *p* = 0.001; Supplementary Table S1). This association remained after adjustment for age (beta coefficient 0.758; *p* = 0.045), suggesting a possible explanation for poorer visual outcomes among patients with *RS1* null genotypes.

## 4. Discussion

Development of demonstrably efficacious therapies for IRDs has proven challenging due to the typically slow progression of disease and limited understanding of the natural histories of different IRD entities. In addition, there are limited data on the important prognostic clinical findings of IRDs due to their rarity in clinical practice and seemingly variable presentations despite shared genotypes. Here, we have used a cohort of XLRS patients to elucidate features of disease that clinicians may use for prognosis and clinical trialists may use to improve chances of demonstrating efficacy over the typically brief course of an interventional trial.

One common finding among published XLRS patient cohorts is the slow progression of vision loss (4, 6, 24, 25, 29, 30), which has important implications for the assessment of treatment responses in a clinical trial. We found no decline in median acuity over a median follow-up of 7 years, and a mean acuity decline only equivalent to 1.57 ETDRS letters per year, which is well within the error margin of routine clinical testing. A previous systematic review of XLRS cohort studies (6) demonstrated an average annual decline of 0.22–0.5 ETDRS letters. Despite this decline, however, in those studies, as well as a more recent large cohort study from Belgium and The Netherlands (30), an initial increase in VA was observed in the first to second decade of life before a slow decline phase. This trend of visual acuity increase was not observed in our data, although we did observe progression of outer retinal atrophy over the first two decades of life, with atrophy severity correlating with worsened visual acuity. A smaller US-based cohort study (36) similarly demonstrated that outer segment thickness but not total retinal thickness correlated with visual acuity, in agreement with our current findings. The relative stability of VA through multiple years of life highlights the challenge of using VA as a primary trial endpoint, as well as the importance of patient selection for potential treatment trials.

Inter-eye disease symmetry is a cardinal feature of most IRDs, and this feature is essential for the conduct of interventional trials that utilize untreated fellow eyes as an untreated control. In XLRS, prior studies have suggested good inter-eye correlation using CST as the outcome measure: for example, in one study of 120 patients, an *r*-squared value of 0.83 was observed, compared to an *r*-squared value of 0.47 for VA (37). However, our data show greater inter-eye variability than previously observed. Using the same outcome measures, we obtained *r*-squared values of 0.146 and 0.029 for inter-eye CST and VA, respectively. Only CST was statistically significant, but this exhibited greater deviation from linearity compared to the previous study, probably due to the smaller sample size used in the current study. It follows that interventional clinical trials involving only tens of patients, such as those mentioned previously (14, 23), would be highly susceptible to inter-eye variability confounding the results if a fellow eye control study design is used. Although a fellow eye comparator design is still likely optimal because it minimizes the risks to the untreated fellow eye from the experimental therapy, a large patient number and long follow-up would be required with such a design to demonstrate clinical benefit.

Due to the challenges with VA as a clinical trial endpoint, including accurate quantitative measurement of intra-eye variations in CST, ORA, or related structural measurements will be valuable to consider for trial design. For ORA, however, consideration needs to be given to the extent of ORA at the commencement of the trial, as it seems unlikely that restoration of normal *RS1* function would reverse pre-existing photoreceptor loss. Moreover, our observation that ORA continued to develop in a patient despite resolution of visible foveoschisis on OCT suggests that it may not be possible to halt progression of ORA once it begins. Therefore, to maximize structural preservation, XLRS trials should prioritize enrollment of patients with intact outer retinal structure at baseline, which based on our findings would require patients predominantly under 20 years of age. In addition to structural outcomes, it is likely that additional functional outcomes will be essential to demonstrate therapeutic efficacy in XLRS treatment trials. The abnormal responses of XLRS patients on microperimetry, static perimetry, electroretinography, and pupillometry are well established and have been proposed for use as trial endpoints (38–41). In a recently reported AAV2-mediated *RS1* gene augmentation trial, clinical response at 12 months was demonstrated only for two endpoints: static perimetry, with two responders of 25 participants, and reduction in cyst cavity volume on SD-OCT, with one responder of 23 participants (23). Despite these limited responses, it is likely that further optimization of both structural and functional endpoints will enable demonstration of therapeutic efficacy in future trials.

Additionally, we acknowledge that there may be additional mechanisms of visual acuity loss in XLRS patients that were not detected in the present study. We did not observe the development of frank optic atrophy in our cohort, but it is conceivable that chronic inner retinal schisis of the macula may lead to damage of the nerve fiber layer and progressive loss of visual acuity. Glaucoma has been found in association with macular schisis in several reports (42, 43), although we are unaware of a study that has investigated this systematically in XLRS patients via OCT of the nerve fiber layer. It may be useful to explore this possibility in currently available XLRS patient cohorts as an additional prognostic factor for vision loss.

Contemporary interventions for XLRS remain limited to management of macular schisis with topical or systemic CAIs (44–46) (VA and CST improve for 2 years then wane), and vitreoretinal surgery in cases of schisis-related RD. (47, 48) We observed a small but significant reduction in CST at 3–6 months following commencement of a topical CAI, consistent with previous reports of CAI anatomical efficacy (46). This was not accompanied by a significant improvement in VA, which is consistent with our finding that CST did not correlate significantly with VA, although we acknowledge that a long-term follow-up may be required to observe the benefits of this intervention. Previous work by Ambrosio and colleagues (46) showed that CAIs offer a transient benefit to VA and CST over 2 years before their effect declines, and we have speculated that the degree of response might be related in part to integrity of deep capillary plexus vasculature (34). We speculate that long-term study of CAI use in XLRS patient may demonstrate efficacy in the form of slowed ORA secondary to CST reduction, although this effect is likely to be modest. Future longitudinal studies using OCT angiography (which was not included in this study) might be informative.

RD is a relatively common complication of XLRS, reported to occur in 0–23% of cases (24, 49). Similarly, we observed RD in 7.7% of eyes in our series, all but one of which underwent vitrectomy with stable retinal findings post-operatively. Perhaps unsurprisingly, peripheral retinoschisis was seen in all eyes with RD, and this was observed more often than in eyes without RD. No eyes in our series were managed with vitrectomy in the absence of RD, although previous work from Japan has suggested than this may decrease the risk of RD in XLRS cases with severe peripheral retinoschisis (50). The same group suggested that foveoschisis may be related to abnormal vitreo-retinal adherence (50), although current literature suggests that retinoschisin is primarily involved in intra-retinal, intercellular adhesion via plasma membrane-bound Na/K-ATPase (20, 51–53). Clinically, our data suggest that XLRS patients with peripheral schisis be counselled about their heightened risk for RD compared to patients without peripheral involvement, but at present the weight of evidence does not support the use of prophylactic vitrectomy in XLRS patients.

In our cohort, a strong association between null *RS1* genotypes and moderate or worse visual impairment at the final visit was demonstrated, which was independent of presenting structural features or the age at symptom onset. Potential phenotype–genotype correlations have been suggested previously for XLRS. An electrophysiological study by Vincent and colleagues from the United Kingdom (26) demonstrated that nonsense, splice-site, and frame-shift mutations in *RS1* were associated with electronegative ERGs, delayed flicker responses, and abnormal pattern ERGs. Missense mutations in the same study demonstrated more variable ERG responses, consistent with variation in residual *RS1* function. In contrast, several groups were unable to demonstrate associations between XLRS disease severity and genotype, the first being a UK study of 86 XLRS patients (25). That study examined visual acuity and structural changes among patients with truncating and missense *RS1* mutations but did not find significant differences between the groups, although supporting data were not shown in the final article. A recent large cohort study (30) of 340 XLRS patients from The Netherlands and Belgium did not identify any clear phenotype–genotype correlations linking the severity of *RS1* mutations to the clinical severity of disease, although again supporting data were not shown in the article. A Spanish cohort study of XLRS found that severe genotypes were not correlated with RD, VH, or strabismus, while VA was not included as an outcome measure (54). Another paper, based on study of a Chinese cohort of XLRS patients, found that poor visual outcomes tended to associate with null *RS1* genotypes (55). We used the cutoff of moderate visual impairment (Snellen 20/63 or logMAR 0.5) or worse as a clinically relevant outcome measure with which to establish a phenotype–genotype correlation because this represents the median visual acuity of XRLS patients in both this and prior cohort studies (7, 30), enabling separation of patients into two relatively evenly-sized groups of visual acuity. Patients with null mutations had 7.8-fold the odds of at least moderate visual impairment at last follow-up, regardless of their initial age at symptom onset. Our additional finding that more extensive ORA occurred more often among null *RS1* genotype eyes also hints at an explanation for this correlation. It will be interesting to determine if a null genotype has similarly strong predictive power for functional outcomes when applied to other regional cohorts.

To date there have been two phase 1/2 gene therapy trials for XLRS, both involving intravitreal AAV-mediated *RS1* gene augmentation (14, 23). Despite these pioneering efforts, neither trial demonstrated clinically meaningful improvements to retinal function as measured by VA, perimetry, OCT, or electrophysiology. Moreover, both trials had cases of intraocular inflammation in the form of anterior and/or posterior uveitis, though this was generally manageable with systemic steroids. In light of data presented in the current work and elsewhere (6, 9, 25, 30, 37), we suggest that the natural history of XLRS may preclude observation of a clinically meaningful outcome in the brief 12–24 months observation window of a conventional clinical trial. The low average rate of visual decline (<2 ETDRS letters/year), presumably high incidence of amblyopia among XLRS patients, inter-eye asymmetry, and minimal change in macular thickness over long observation periods, should prompt a re-thinking of how an interventional XLRS treatment trial should be designed. Using contemporary structural and functional outcome measures, we suggest that a more prolonged trial period (likely greater than 5 years) will be necessary to observe stabilization of the disease unless patients are enrolled during their visual acuity decline phase, in which case a briefer trial may be possible. A more optimally designed trial would involve young patients with null genotypes, intact outer retinal structure, and normal or near-normal acuity at trial commencement. This may increase the chance of trial success, compared to the phenotypically and genotypically heterogeneous patient groups that are typically used for IRD treatment trials.

## Data availability statement

The original contributions presented in the study are included in the article/Supplementary material, further inquiries can be directed to the corresponding author.

## Ethics statement

The studies involving human participants were reviewed and approved by University of Iowa Institutional Review Board. Written informed consent to participate in this study was provided by the participants’ legal guardian/next of kin.

## Author contributions

BF and IH conceived the study. BF drafted the manuscript and performed the data analysis. JR, ES, SR, HB, ADr, ADu, JA, and ES critically revised the manuscript for intellectual content. All authors contributed to the article and approved the submitted version.

## Funding

This work was supported by Iowa Institute for Vision Research, University of Iowa, Iowa City, Iowa, National Institutes of Health P30-EY025580, Research to Prevent Blindness Unrestricted Grant to the University of Iowa, and Chakraborty Foundation Grant (Drack) and Professorship (Dumitrescu).

## Conflict of interest

The authors declare that the research was conducted in the absence of any commercial or financial relationships that could be construed as a potential conflict of interest.

## Publisher’s note

All claims expressed in this article are solely those of the authors and do not necessarily represent those of their affiliated organizations, or those of the publisher, the editors and the reviewers. Any product that may be evaluated in this article, or claim that may be made by its manufacturer, is not guaranteed or endorsed by the publisher.
