# Supplementary material for: Long-term functional and structural outcomes in X-linked retinoschisis: implications for clinical trials
Source: Front Med (Lausanne). 2023 Jun 15;10:1204095. doi: 10.3389/fmed.2023.1204095 (PMC10310546; doi:10.3389/fmed.2023.1204095)
Supplement: Supplementary file 1 [file Data_Sheet_1.docx]

Supplementary Material

Long-term functional and structural outcomes in X-linked retinoschisis: implications for clinical trials

Beau J. Fenner^1,2,3,4,5^, Jonathan F. Russell^1,2^, Arlene V. Drack^1,2^, Alina V. Dumitrescu^1,2^, Elliott H. Sohn^1,2^, Stephen R. Russell^1,2^, H. Culver Boldt^1,2^, Louisa M. Affatigato^1^, Jeremy M. Hoffmann^1^, Jeaneen L. Andorf^1^, Edwin M. Stone^1,2^, Ian C. Han^1,2*^

^1^Institute for Vision Research, University of Iowa, Iowa City, IA, USA

^2^Department of Ophthalmology and Visual Sciences, Carver College of Medicine, University of Iowa, Iowa City, IA, USA

^3^Department of Medical Retina, Singapore National Eye Centre, Singapore

^4^Singapore Eye Research Institute, Singapore

^5^Ophthalmology and Visual Sciences Academic Clinical Program, Duke-NUS Graduate Medical School, Singapore

*** Correspondence:**Ian C. Han, MD
ian-han@uiowa.edu

**Supplementary Table 1.** Associations between *RS1* genotype and the presence of outer retinal atrophy (ORA) in XLRS patients. ORA grades were compared between patients with *RS1* null and non-null mutations at the last recorded optical coherence tomography (OCT) scan.

|  | **ORA grade** | | | | | | | **Age adjusted model*** |
| --- | --- | --- | --- | --- | --- | --- | --- | --- |
| ***RS1* genotype category** | **0** |  | **1** |  | **2** |  | **3** |  |
| Non-null mutation | 4/40 (10) |  | 8/40 (20) |  | 27/40 (68) |  | 1/40 (3) | Coefficient 0.758 (95% CI 0.017, 1.499); p = 0.045 |
| Null mutation | 0/14 (0) |  | 3/14 (21) |  | 6/14 (43) |  | 5/14 (36) |  |
|  |  |  |  |  |  |  |  |  |
| Significance* | 0.223 |  | 0.936 |  | 0.101 |  | 0.001* |  |

*Statistical significance with p <0.05, using the Chi-squared test of proportions. In the age-adjusted model, logistic regression was performed with adjustment for patient age at the time of the final OCT imaging.

**Supplementary Figure 1.** Relationship between visual acuity and central subfield thickness (CST) on optical coherence tomography in patients with XLRS. Pairwise comparisons between CST subgroups were made using Kruskal-Wallis analysis of variance (ANOVA). Red bars indicate the median CST for each subgroup. Ns, not significant (p ≥0.05).

**Supplementary Figure 2.** Relationship between central subfield thickness (CST) and severity of outer retinal atrophy (ORA) in patients with XLRS. Pairwise comparisons between ORA grades were made using Kruskal-Wallis analysis of variance (ANOVA). Red bars indicate the median CST for each subgroup. Ns, not significant (p ≥0.05); **, significant with p = 0.0036.
